# Ginsenoside Rg3 Improves Recovery from Spinal Cord Injury in Rats via Suppression of Neuronal Apoptosis, Pro-Inflammatory Mediators, and Microglial Activation

**DOI:** 10.3390/molecules22010122

**Published:** 2017-01-12

**Authors:** Dong-Kyu Kim, Ki-Jung Kweon, Pyungsoo Kim, Hee-Jung Kim, Sung-Soo Kim, Nak-Won Sohn, Sungho Maeng, Jung-Won Shin

**Affiliations:** Department of East-West Medical Science, Graduate School of East-West Medical Science, Kyung Hee University, Yongin 17404, Korea; omdkdk@empal.com (D.-K.K.); kgj1031@hanmail.net (K.-J.K.); bixas@hanmail.net (P.K.); noonkkot@hanmail.net (H.-J.K.); kokoajam@gmail.com (S.-S.K.); sohnnw@khu.ac.kr (N.-W.S.)

**Keywords:** ginsenoside Rg3, spinal cord injury, motor function, apoptosis, pro-inflammatory cytokines, microglial activation

## Abstract

Spinal cord injury (SCI) is one of the most devastating medical conditions; however, currently, there are no effective pharmacological interventions for SCI. Ginsenoside Rg3 (GRg3) is one of the protopanaxadiols that show anti-inflammatory, anti-oxidant, and neuroprotective effects. The present study investigated the neuroprotective effect of GRg3 following SCI in rats. SCI was induced using a static compression model at vertebral thoracic level 10 for 5 min. GRg3 was administrated orally at a dose of 10 or 30 mg/kg/day for 14 days after the SCI. GRg3 (30 mg/kg) treatment markedly improved behavioral motor functions, restored lesion size, preserved motor neurons in the spinal tissue, reduced Bax expression and number of TUNEL-positive cells, and suppressed mRNA expression of pro-inflammatory cytokines including tumor necrosis factor-α, interleukin (IL)-1β, and IL-6. GRg3 also attenuated the over-production of cyclooxygenase-2 and inducible nitric oxide synthase after SCI. Moreover, GRg3 markedly suppressed microglial activation in the spinal tissue. In conclusion, GRg3 treatment led to a remarkable recovery of motor function and a reduction in spinal tissue damage by suppressing neuronal apoptosis and inflammatory responses after SCI. These results suggest that GRg3 may be a potential therapeutic agent for the treatment of SCI.

## 1. Introduction

Spinal cord injury (SCI) is one of the most devastating medical conditions that can temporarily or permanently impair physical functions. SCI is characterized by an initial physical damage (primary injury) leading to one or more progressive damaging processes (secondary injury) that spread away from the injury epicenter [1]. The initial physical injury leads to tissue necrosis and a disruption of neuronal and vascular structures. The secondary injury process is predominantly responsible for the damage associated with SCI, including inflammation, oxidative stress, ischemia, necrosis, and neuronal apoptosis [2]. Many studies have investigated a variety of interventions targeting these secondary processes [2,3]. Despite recent advances including transplantation of neural stem cells, tissue engineering, and molecular therapy [4,5], neurological recovery following SCI remains limited, and currently there are no effective pharmacological interventions. However, many aggravating factors associated with the secondary injury may be contemplated as targets for therapeutic interventions.

In recent years, several active ingredients present in medicinal herbs, such as ginsenosides, have attracted much attention in the field of SCI therapeutics [6]. Although ginsenosides, the main active ingredients in *Panax ginseng* C.A. Meyer, are not used to treat SCI regularly, their potential therapeutic effect in SCI treatment has been studied based on their anti-inflammatory, antioxidant, anti-apoptotic, and neuroprotective actions [7,8,9,10,11]. Crude extracts of ginseng improved the recovery of motor function after SCI by inhibiting post-traumatic inflammatory responses [7,8]. Ginsenoside Rb1 (GRb1) inhibited neuronal apoptosis and tissue damage by enhancing spinal aquaporin-4 expression and improved neurological deficits following spinal cord ischemia-reperfusion injury in rats [9]. Ginsenoside Rd (GRd) inhibited the activation of mitogen-activated protein kinase (MAPK) signaling pathway induced by SCI, and consequently improved the locomotor function of rats after SCI through its anti-inflammatory and anti-apoptotic actions [10,11].

**Figure 1:**
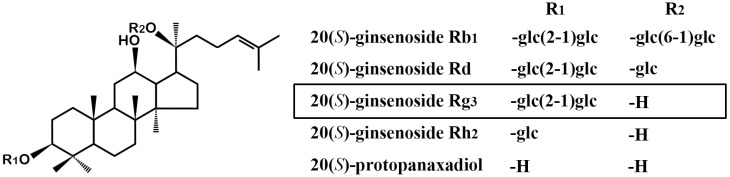
Chemical structures of ginsenosides and their related compounds.

Ginsenoside Rg3 (GRg3) is a dammarane-type tetracyclic terpene sapogenin classified as protopanaxadiol that contains Rb1, Rd, and Rh2 found in ginseng (Figure 1), and is one of the most effective ginsenosides that showed anti-inflammatory, antioxidant, and neuroprotective effects in various in vitro and in vivo studies [12]. GRg3 decreased the expression of pro-inflammatory mediators such as tumor necrosis factor-α (TNF-α), interleukin (IL)-1β, IL-6, and cyclooxygenase-2 (COX-2) [13,14,15], and inhibited the overproduction of inducible nitric oxide synthase (iNOS) [16]. GRg3 also exerted neuroprotective action through enhancement of the free radical scavenging [17], attenuation of microglial activation in the brain [18], and inhibition of *N*-methyl-d-aspartate (NMDA) receptors and L-type Ca^++^ channels [19,20]. Moreover, GRg3 epimers, 20(*S*)- and 20(*R*)-, showed remarkable neuroprotective effects such as improving neurological outcomes and reducing the infarct size following cerebral ischemia-induced brain injury in vivo [21,22]. From these previous results, it was hypothesized that GRg3 may also be beneficial for SCI. Therefore, we investigated the neuroprotective effects of GRg3 following SCI. We evaluated the improvement of motor function and spinal tissue damage and examined the effects on neuronal apoptosis, pro-inflammatory mediators, and microglial activation following the treatment with GRg3 after compressive SCI in rats.

## 2. Results

### 2.1. GRg3 Improves Behavioral Motor Functions of Rats Subjected to Compressive SCI

**Figure 2:**
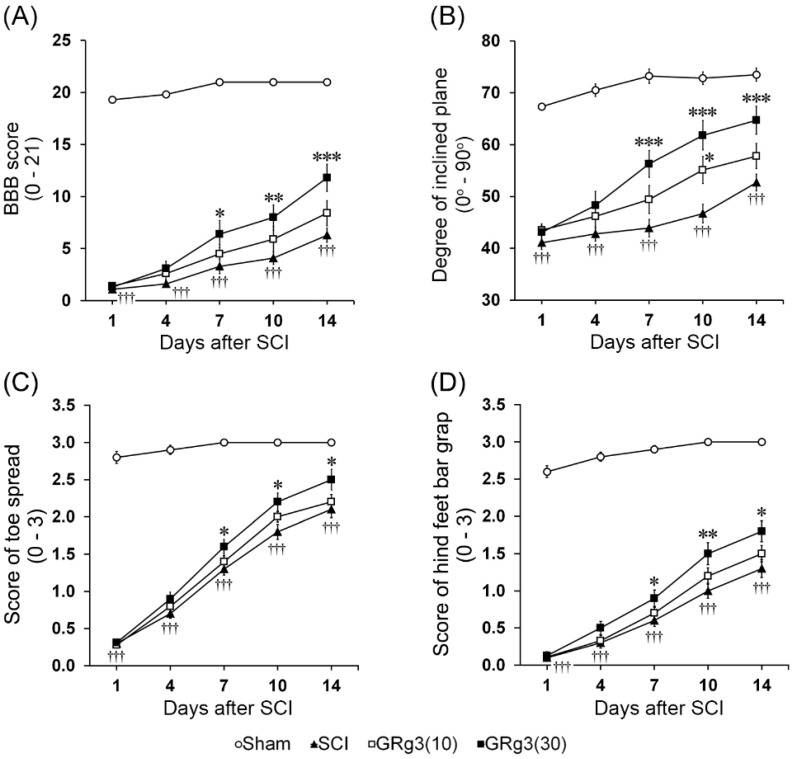
Effect of Ginsenoside Rg3 (GRg3) on hindlimb behavioral motor functions of rats subjected to spinal cord injury (SCI). GRg3 (30 mg/kg) group showed a significant functional recovery from SCI in the Basso-Beattie-Bresnahan (BBB) locomotor rating (**A**); the inclined plane test (**B**); the toe spread test (**C**); and the hind foot bar grab test (**D**) starting at one week following SCI. Data are represented as mean ± SEM (*n* = 18 rats in each group; ††† *p* < 0.001 between Sham and SCI group; * *p* < 0.05, ** *p* < 0.01, *** *p* < 0.001 between SCI and GRg3 groups).

Hindlimb behavioral motor functions were significantly impaired in the SCI group. Immediately after compression injury, the rats in all groups showed paralysis of both hindlimbs. Scores for the Basso-Beattie-Bresnahan (BBB) rating, the toe spread, and the hind foot bar grab test were 0, and the maximum degree in the inclined plane test was ~40°. Although motor functions gradually improved during 14 days of the experiment, the scores of behavioral motor function tests were significantly lower (*p* < 0.001) than those of the sham operated group (Figure 2A–D). The treatment with 30 mg/kg of GRg3 led to significant elevation in scores of the BBB rating, the inclined plane test, the toe spread test, and the hind foot bar grab test starting at one week following SCI (Figure 2A–D). The recovery of behavioral motor functions was accelerated starting at one week after GRg3 treatment. At the 14th day after compression injury, most rats in the SCI group showed minimal movement of two joints and no plantar stepping, partial toe spreading, and hind foot responses to a contact with the bar, without being able to grab it. Contrarily, the rats treated with 30 mg/kg of GRg3 showed occasional weight-supported plantar steps and frequent forelimb-hindlimb coordination. The toe spreading improved more than partial spreading state, and they were able to grab the bar with the hind foot. The maximum degree in the inclined plane test reached up to 60°. Compared to the SCI group, GRg3(30) group demonstrated significant increases in scores of the BBB rating (11.8 ± 1.3 vs. 6.3 ± 0.7, *p* < 0.001; Figure 2A), the inclined plane test (64.7° ± 2.7° vs. 52.7° ± 1.6°, *p* < 0.001; Figure 2B), the toe spread test (2.5 ± 0.1 vs. 2.1 ± 0.1, *p* < 0.05; Figure 2C), and the hind foot bar grab test (1.8 ± 0.1 vs. 1.3 ± 0.1, *p* < 0.05; Figure 2D).

### 2.2. GRg3 Reduces Spinal Tissue Damage of Rats Subjected to Compressive SCI

**Figure 3:**
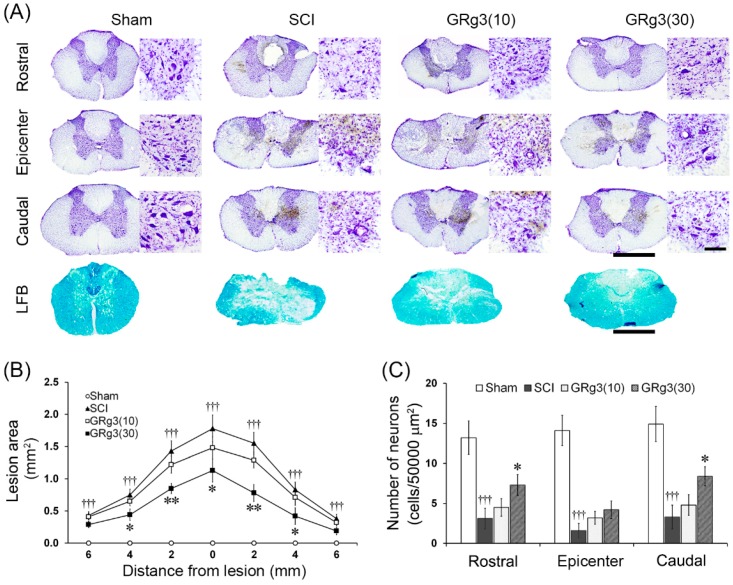
Effects of GRg3 on histological changes in the spinal cord of rats subjected to spinal cord injury (SCI). (**A**) Representative photographs showing spinal cord sections at a level of the lesion epicenter, at 4 mm rostral to, and at 4 mm caudal to the lesion epicenter stained using cresyl violet or Luxol fast blue (LFB) method (scale bar = 1 mm). Photographs taken at higher magnification show motor neurons in the ventral horn (scale bar = 100 μm). Photographs of LFB stained sections show spared white matter at the level of the lesion epicenter (scale bar = 1 mm); (**B**) GRg3 (30 mg/kg) group showed a significant attenuation in the lesion area; (**C**) GRg3 (30 mg/kg) group showed a significant increase in number of spared neurons in the ventral horn in rostral and caudal regions, but not in the lesion epicenter. Data are represented as mean ± SEM (*n* = 6 in each group; ††† *p* < 0.001 between Sham and SCI group; * *p* < 0.05, ** *p* < 0.01 between SCI and GRg3 groups).

The spinal tissue of SCI rats showed severe histological damage in the gray and white matter. Even after 14 days, SCI rats demonstrated foci of cystic necrosis in the dorsal area and hemorrhage, vacuolations, deformations, and disappearance or shrinkage of motor neurons in the ventral horn gray matter. In the lesion epicenter, general features of histological outcomes in the GRg3 treatment groups were not different from the SCI group (Figure 3A). After 14 days of GRg3 treatment, 30 mg/kg of GRg3 treatment group showed a significant reduction (*p* < 0.05 or *p* < 0.01) in the size of the lesion area measured on LFB-stained sections at 2 mm intervals (Figure 3B). The number of spared motor neurons counted on CV-stained sections was significantly reduced (*p* < 0.001) in the SCI group. The group treated with 30 mg/kg of GRg3 showed a significant restoration (*p* < 0.05) of spared motor neurons in the rostral and caudal segments 4 mm apart from the lesion epicenter, but no difference in the lesion epicenter (Figure 3C).

### 2.3. GRg3 Attenuates Neuronal Apoptosis in the Spinal Cord

**Figure 4:**
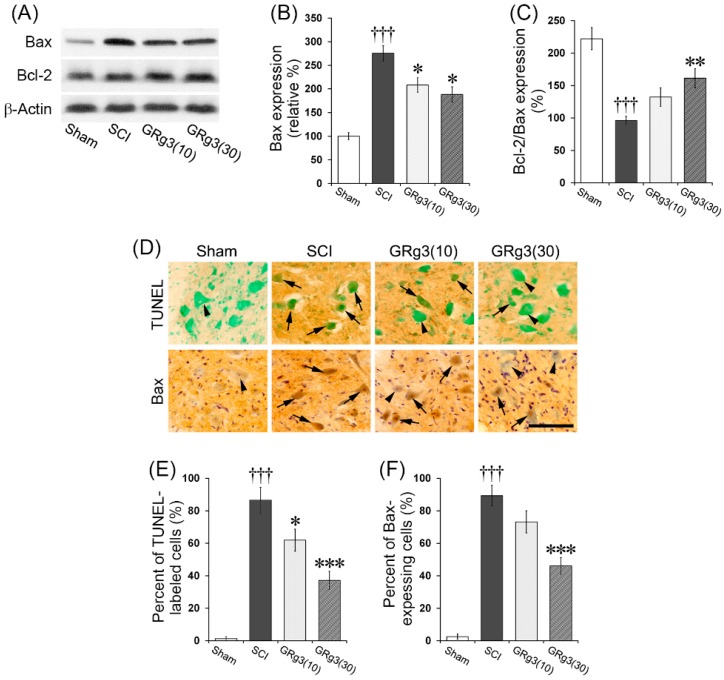
Effects of GRg3 on neuronal apoptosis in the spinal cord of rats subjected to spinal cord injury (SCI). (**A**) Representative western blots illustrating the differences in band intensities of Bax and Bcl-2; (**B**) GRg3 treatment at doses of 10 mg/kg and 30 mg/kg significantly attenuated the upregulation of Bax expression; (**C**) GRg3 treatment at a dose of significantly increased the reduction of the ratio of Bcl-2 per Bax expression; (**D**) Representative photographs showing spinal cord sections immuno-stained with TUNEL and Bax (scale bar = 100 μm). Arrow heads indicate TUNEL- or Bax-negative neurons and arrows indicate TUNEL- or Bax-positive neurons; (**E**) GRg3 treatment at doses of 10 mg/kg and 30 mg/kg significantly reduced the percentage of TUNEL-labeled neurons in the ventral horn; (**F**) GRg3 treatment at a dose of 30 mg/kg significantly reduced the percentage of Bax-expressing neurons in the ventral horn. Data are represented by mean ± SEM (*n* = 6 in each group; ††† *p* < 0.001 between Sham and SCI group; * *p* < 0.05, ** *p* < 0.01, *** *p* < 0.001 between SCI and GRg3 groups).

As observed by western blot analyses, the SCI group showed a significant increase (*p* < 0.001) in Bax expression in the spinal tissue, while the GRg3 groups showed a significant reduction (*p* < 0.05) compared to the SCI group (Figure 4A,B). Although Bcl-2 expression in the spinal tissue was not different among the groups, the ratio of Bcl-2/Bax expression was significantly lower (*p* < 0.001) in the SCI group and significantly elevated (*p* < 0.01) in the 30 mg/kg GRg3 group (Figure 4A,C). In SCI rats, shrinking of the cell body was observed in most TUNEL-positive cells, and the percentage of TUNEL-positive cells in the ventral horn gray matter was more than 85%. The GRg3 treatment significantly reduced the percentage of TUNEL-positive cells to ~62% (*p* < 0.05) in the 10 mg/kg dose group and to ~37% (*p* < 0.001) in the 30 mg/kg dose group (Figure 4D,E). SCI also increased the percentage of Bax-expressing cells in the ventral horn gray matter to ~90%, while GRg3 treatment significantly reduced it to ~46% (*p* < 0.001) in the 30 mg/kg dose group, while no differences were observed in the 10 mg/kg dose group (Figure 4D,F).

### 2.4. GRg3 Attenuates TNF-α, IL-1β, and IL-6 mRNA Expression in the Spinal Cord

**Figure 5:**
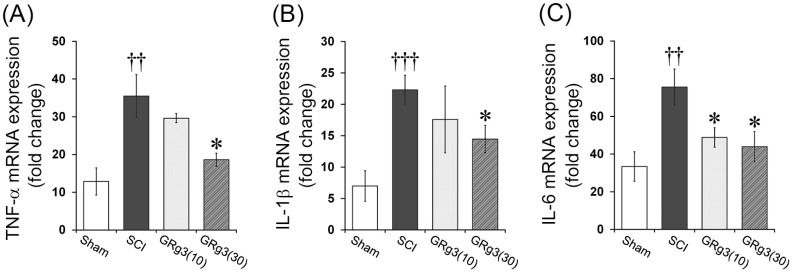
Effects of GRg3 on pro-inflammatory cytokine expression in the spinal cord of rats subjected to spinal cord injury (SCI). GRg3 treatment significantly attenuated the upregulation of TNF-α mRNA expression (**A**); IL-1β mRNA expression (**B**); and IL-6 mRNA expression (**C**) at a dose of 30 mg/kg. Data are represented as mean ± SEM (*n* = 6 rats in each group; †† *p* < 0.01, ††† *p* < 0.001 between Sham and SCI group; * *p* < 0.05 between SCI and GRg3 groups).

Levels of mRNA expression of pro-inflammatory cytokines (TNF-α, IL-1β, and IL-6) in the spinal tissue were measured by qRT-PCR. The Sham operated group showed an increase in pro-inflammatory cytokine expression, solely due to the laminectomy. The SCI group showed a significant increase in TNF-α (*p* < 0.01), IL-1β (*p* < 0.001), and IL-6 (*p* < 0.01) mRNA expression levels in the spinal tissue compared to the Sham group. The treatment with 30 mg/kg of GRg3 significantly attenuated TNF-α, IL-1β, and IL-6 mRNA expression (*p* < 0.05, respectively), while the treatment with 10 mg/kg of GRg3 significantly attenuated only IL-6 mRNA expression (*p* < 0.05) compared to the SCI group (Figure 5A–C).

### 2.5. GRg3 Attenuates iNOS and COX-2 Expressions in the Spinal Cord

**Figure 6:**
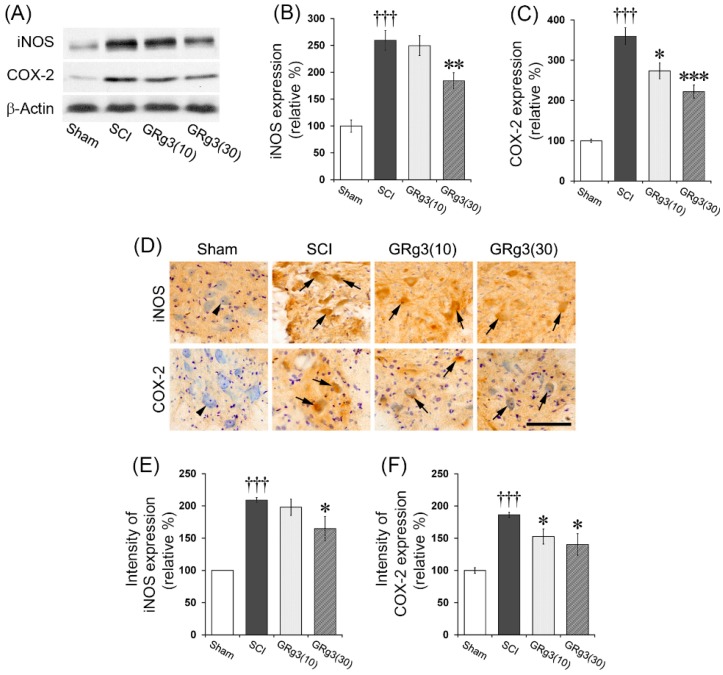
Effects of GRg3 on inducible nitric oxide synthase (iNOS) and cyclooxygenase-2 (COX-2) expression in the spinal cord of rats subjected to spinal cord injury (SCI). (**A**) Representative western blots illustrating the differences in band intensities of iNOS and COX-2; (**B**) GRg3 treatment significantly attenuated the upregulation of iNOS expression at a dose of 30 mg/kg; (**C**) GRg3 treatment significantly attenuated the upregulation of COX-2 expression at doses of 10 and 30 mg/kg; (**D**) Representative photographs showing spinal cord sections immuno-stained for iNOS and COX-2 (scale bar = 100 μm). Arrows indicate iNOS- or COX-2-expressing neurons; (**E**) GRg3 treatment significantly reduced the intensity of iNOS expression in the neurons at a dose of 30 mg/kg; (**F**) GRg3 treatment significantly reduced the intensity of COX-2 expression in the neurons at doses of 10 and 30 mg/kg. Data are represented by mean ± SEM (*n* = 6 rats in each group; ††† *p* < 0.001 between Sham and SCI group; * *p* < 0.05, ** *p* < 0.01, *** *p* < 0.001 between SCI and GRg3 groups).

The SCI group showed a significant increase (*p* < 0.001) in the expression levels of iNOS in the spinal tissue, while the treatment with 30 mg/kg of GRg3 led to significant reduction in these levels (*p* < 0.01) compared to the SCI group (Figure 6A,B). The SCI group also showed a significant increase (*p* < 0.001) in COX-2 expression in the spinal tissue, while the GRg3 treatment group showed a significant reduction (10 mg/kg dose, *p* < 0.05; 30 mg/kg dose, *p* < 0.001) compared to the SCI group (Figure 6A,C). The immunohistochemical analyses of iNOS and COX-2 expression showed a similar pattern of changes. In the SCI group, intensities of iNOS and COX-2 expression in the motor neurons significantly increased; however, in the 30 mg/kg dose group, intensities of iNOS (*p* < 0.05) and COX-2 (*p* < 0.05) expression significantly attenuated (Figure 6D–F).

### 2.6. GRg3 Suppresses Microglial Activation in the Spinal Cord

**Figure 7:**
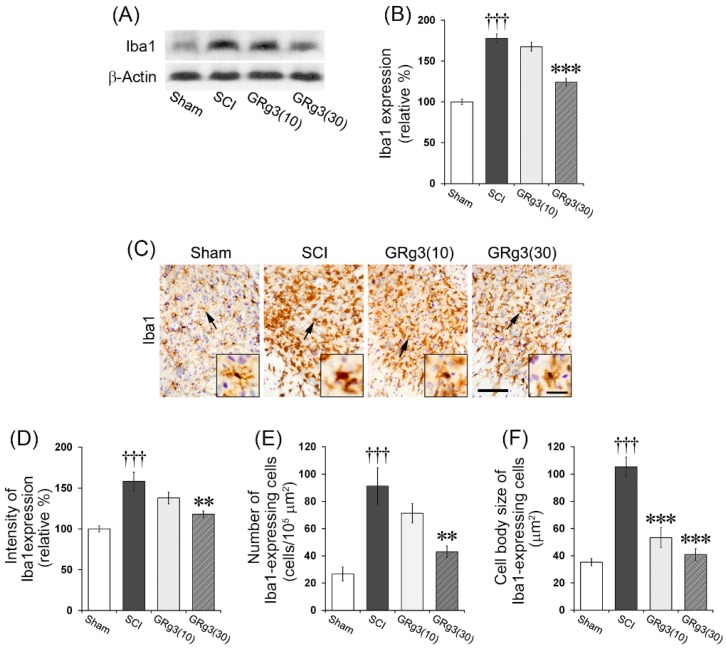
Effects of GRg3 on microglial activation in the spinal cord of rats subjected to spinal cord injury (SCI). (**A**) Representative western blots illustrating the differences in band intensities of anti-ionized calcium binding adaptor molecule 1 (Iba1); (**B**) GRg3 treatment significantly attenuated the upregulation of Iba1 expression at a dose of 30 mg/kg; (**C**) Representative photographs showing spinal cord sections immuno-stained for Iba1 (scale bar = 100 μm). Arrows indicate Iba1-expressing microglia. Photographs taken at higher magnification show morphological changes of activated microglia (scale bar = 25 μm); (**D**) GRg3 treatment significantly reduced the intensity of Iba1 expression at a dose of 30 mg/kg; (**E**) GRg3 treatment significantly attenuated the numbers of Iba1-expressing microglia in the spinal cord at a dose of 30 mg/kg; (**F**) GRg3 treatment significantly reduced the cell body size of Iba1-expressing microglia at doses of 10 and 30 mg/kg. Data are represented as mean ± SEM (*n* = 6 rats in each group; ††† *p* < 0.001 between Sham and SCI group; ** *p* < 0.01, *** *p* < 0.001 between SCI and GRg3 groups).

In order to elucidate the effect of GRg3 on the inflammatory response following SCI, the effect on microglial activation in the spinal tissue was evaluated. The SCI group showed a significant increase (*p* < 0.001) in the expression of Iba1 (a marker of microglial activation) in the spinal tissue, while the 30 mg/kg dose group showed significant reduction (*p* < 0.001) in Iba1 expression compared to the SCI group (Figure 7A,B). Immunohistochemical analyses using Iba1 antibody on the spinal tissue of SCI group demonstrated that most of the microglia in the lesion epicenter showed activated and phagocytic phenotype consisting of large, amoeboid-shaped cell body without processes. Microglia in the perilesional segments showed a phenotype suggestive of activation as they exhibited increased cell size, irregular shape, thickened and shortened processes, and intensified Iba1 immuno-staining density (Figure 7C). Microglial activation was predominant in the gray matter. In the lesion epicenter, morphological features of the microglia in the GRg3 treated group were similar to those in the SCI group. In the perilesional segments, the GRg3 (30 mg/kg) treated group showed a significant reduction in the Iba1 expression intensity (*p* < 0.01), the number (*p* < 0.01), and the cell body size (*p* < 0.001) of Iba1-expressing microglia compared to those of the SCI group (Figure 7D–F).

## 3. Discussion

In this study, we examined the neuroprotective effects of GRg3 in rats with compressive SCI. GRg3 (30 mg/kg) promoted the recovery of motor function, ameliorated spinal tissue damage, attenuated SCI-induced neuronal apoptosis, inhibited the overproduction of pro-inflammatory mediators, and suppressed microglial activation. Thus, this study shows that GRg3 has neuroprotective and anti-inflammatory effects on SCI, and consequently improving the functional outcomes.

Assessing the impairment of behavioral motor functions in experimental animal models of SCI is essential to investigate potential therapies for SCI [23]. The present results show that GRg3 treatment enhanced locomotor recovery as compared to the SCI group. The body weight of all the rats in the SCI, GRg3 (10), and GRg3 (30) groups decreased significantly during the first 3 days after SCI. After that, it gradually recovered (Figure S1). A significant difference in behavioral motor functions between the GRg3 group and the SCI group was shown starting at one week following GRg3 treatment. At 14 days following GRg3 treatment, behavioral motor functions markedly improved as compared to the SCI group. GRg3-treated rats exhibited a faster and greater degree of recovery in coordinated hindlimb use while maintaining their body position. There have been no previous reports to study the effect of GRg3 on behavioral motor functions after SCI in vivo. Recently, it has been reported that treatment with GRb1 and GRd, the ginsenosides belonging to the same protopanaxadiol group as GRg3, led to a significant increase in BBB rating scores in spinal cord ischemia-reperfusion injury and spinal contusion injury [9,10,11]. These results indicate that GRg3 has a positive effect on behavioral motor functions after SCI.

Extensive reports on SCI modeling in the rat indicate that the extent of functional impairment is directly related to the severity of histological damage in the spinal cord [24,25]. Secondary injury starts to develop within minutes to hours after primary injury. The secondary injury extends the lesion far beyond the primary damage site, damaging a much larger neuronal area and exacerbating the functional deficits [2,3]. The present study showed that at 14 days following SCI, the lesion size was significantly reduced in the rats treated with GRg3. The number of spared motor neurons in the ventral horn was also significantly restored by the GRg3 treatment. Such sparing of spinal tissue and motor neurons provides a preliminary evidence of functional recovery induced by GRg3 treatment. As the present histological assessments are preliminary and were performed at a light microscopy level, advanced systematic histological analyses at higher magnifications will be necessary for better understanding of the effects induced by GRg3 treatment. However, the present results indicate that GRg3 potentially stimulates histological recovery from SCI.

Apoptosis is an important mediator of secondary injury after SCI. There are at least two stages of secondary injury that involve cellular apoptosis: an initial stage in which necrotic cell death at the lesion epicenter is accompanied by apoptosis of multiple cell types, and a latter phase confined predominantly to the white matter that involves oligodendrocytes [26]. Apoptosis initially occurs at 6 h after injury at the lesion epicenter, and for several days thereafter, the number of apoptotic cells rises steadily. The long-term deficits in motor function after SCI were considered to be a result of widespread apoptosis of neurons and oligodendrocytes in regions distant from the primary injury site [27]. In the present study, GRg3 treatment significantly decreased the number of TUNEL-positive neurons, attenuated the Bax expression, and upregulated the ratio of Bcl-2/Bax expression. In previous studies, it has been reported that GRg3 attenuated apoptosis by inhibiting mitochondrial caspase pathway [28] and Bcl-2/Bax pathway [29]. Taken together with previous reports, the present results suggest that GRg3 exerts protective effects in models of SCI through anti-apoptotic action.

Many mechanisms are involved in the progressive spread of secondary injury after SCI. Inflammatory responses are well known as a major component of secondary injury [3]. Pro-inflammatory cytokines such as TNF-α, IL-1β, and IL-6 play a major role in the initiation and aggravation of secondary injury [30]. TNF-α and IL-1β are implicated in edema formation, leukocyte infiltration, neuronal apoptosis, glial activation, and induction of COX-2 and iNOS [31]. Nitric oxide (NO) and COX-2 also contribute to secondary injury process after SCI [32]. Application of iNOS inhibitor improves behavioral motor functions after SCI [33]. COX-2 inhibitors also improve functional recovery after SCI [34]. Therefore, regulation of these pro-inflammatory mediators is an important target for therapeutic intervention for SCI. In the present study, treatment with GRg3 led to a remarkable reduction in TNF-α, IL-1β, and IL-6 mRNA expression and markedly attenuated the over-expression of iNOS and COX-2 after SCI. These results suggest that GRg3 may be considered as a potential anti-inflammatory agent for the treatment of SCI.

Although microglia exert both beneficial and harmful effects in CNS injury, they also play an important role in secondary injury process after SCI [35]. Microglia are the primary source of the pro-inflammatory cytokines, including TNF-α, IL-1β, and IL-6. These cytokines recruit and activate additional microglial cells. Activated microglia extensively express and release pro-inflammatory cytokines, and consequently extend and exacerbate the inflammatory responses [36]. Furthermore, activation of microglia through Toll-like receptors induces neuronal cell death and neurite degeneration [37]. Microglial activation can also cause axonal retraction through direct interactions [38]. These reports indicate that microglial activation strongly contributes to functional deficits in the injured spinal cord. Treatments aimed at reducing microglial activation are often effective in protecting against SCI [39]. We previously reported that GRg3 has an attenuating effect against microglial activation induced by systemic treatment with lipopolysaccharide in the mouse brain [18]. In the present study, GRg3 also led to remarkable attenuation in Iba1 expression, a marker for detecting activated microglia [40], and diminished the morphological changes characteristic of microglial activation such as increased cell size, irregular shape, and thickened and shortened processes. The present results suggest that GRg3 protects the spinal tissue from secondary injury through the attenuation of microglial activation.

In previous reports, Wang et al. [10] studied the effects of GRd (6, 25, 12.5, 25, or 50 mg/kg/day for 7 days) on spinal cord ischemia/reperfusion injury in rats. They reported that the optimal dose of GRd was 25 mg/kg per day and that the optimal time point was 5 days after ischemia/reperfusion. They also reported that GRd inhibited the expression of pro-apoptotic caspase 3 and downregulated the expression of the apoptotic proteins ASK1 and JNK after ischemia/reperfusion injury in the spinal cord of rats. Cong and Chen [11] also studied the effects of GRd (12.5, 25, or 50 mg/kg/day for 14 days) on spinal cord ischemia/reperfusion injury in rats. They administered GRd (25 and 50 mg/kg/day) 1 week after SCI and found that it significantly improved locomotor function, decreased malondialdehyde levels, increased superoxide dismutase activity, reduced the production of pro-inflammatory cytokines, and prevented cell apoptosis. In addition, Huang et al. [9] studied the effects of GRb1 (10 mg/kg/day for 7 days) on spinal cord ischemia/reperfusion injury in rats. The GRb1 treatment resulted in a significant recovery in behavioral function in a BBB test and a reduction in TUNEL-positive apoptotic cells and aquaporin 4 expression in the spinal cord. The present study administered GRg3 at doses of 10 or 30 mg/kg/day for 14 days to rats with static compressive spinal cord injury. GRg3 (30 mg/kg/day) led to a remarkable recovery in motor function and a reduction in spinal tissue damage when the treatment was commenced 1 week after SCI. The GRg3 treatment also significantly suppressed neuronal apoptosis and inflammatory responses, including elevations in levels of pro-inflammatory cytokines and microglial activation in the spinal cord. Direct comparisons of the efficacy of GRg3 with GRd and GRb1 are impossible because of the different spinal cord injury methods. However, the present findings and those of the earlier reports indicate that ginsenosides, such as GRg3, GRd, and GRb1, all of which are part of the protopanaxadiol group, have a potential therapeutic effect on SCI.

The present study has some limitations. The clinical efficacy, including the optimal therapeutic dose and optimal treatment time, could not be determined because the study relied on animal experimental conditions. Further studies are required to elucidate the underlying mechanisms involved in the protective effects of GRg3 in SCI, and clinical studies are needed to confirm its safety and validate the findings. However, based on the results of the present study, GRg3 may be a candidate for the development of a therapeutic agent for SCI.

## 4. Materials and Methods

### 4.1. Animals

Male Sprague-Dawley rats (12 week-old, 280–300 g; Nara Biotechnology, Seoul, Korea) were used for this study. All animal protocols were approved by the Ethics Committee for the Care and Use of Laboratory Animals at Kyung Hee University. These animals were housed in plastic cages maintained at constant temperature (22 ± 2 °C) and humidity (55% ± 10%) with 12 h–12 h light-dark conditions. They were provided with free access to food and water before the experiments.

### 4.2. Reagents

20(*S*)-Ginsenoside Rg3 from Panax ginseng (C_42_H_72_O_13_; formula weight, 785.01; purity, 95%~99%) was purchased from Biopurify Phytochemicals (Chengdu, China). Mouse anti-Bax and mouse anti-Bcl-2 antibodies for western blotting were purchased from Santa Cruz Biotechnology (Santa Cruz, CA, USA). Rabbit anti-Bax antibody for immunohistochemistry was purchased from Abcam (Cambridge, UK). TACS 2 TdT-DAB in Situ Apoptosis Detection kit for terminal transferase dUTP nick-end labeling (TUNEL) assay was purchased from Trevigen (Gaithersburg, MD, USA). Mouse anti-iNOS antibody was purchased from BD Biosciences (Laguna Hills, CA, USA). Rabbit anti-COX-2 antibody was purchased from Cayman Chemical (Ann Arbor, MI, USA). Rabbit anti-ionized calcium binding adaptor molecule 1 (Iba1) antibody was purchased from Wako Pure Chemical Industries (Osaka, Japan). Mouse anti-β-actin antibody was purchased from Chemicon International (Temecula, CA, USA). Biotinylated anti-mouse and anti-rabbit secondary antibodies were purchased from Vector Laboratories (Burlingame, CA, USA). The remaining chemicals and reagents used were of analytical grade and were obtained from commercial sources.

### 4.3. Experimental Groups and Drug Treatment

The animals were randomly divided into four groups. The sham group (Sham) underwent laminectomy without compression injury. The control group (SCI) was subjected to spinal cord compression injury as described in the methods section and was only administered with the vehicle, which was normal saline at a volume equal to that of the drug administered to GRg3 group. The GRg3 treatment group was treated with 10 mg/kg (GRg3(10)) or 30 mg/kg (GRg3(30)) of GRg3, dissolved in normal saline, delivered orally once a day for 14 days. In total, 72 rats (*n* = 18 per group) were used in this study. All the rats were included in the behavioral assessment. After the behavioral evaluation on the 14th day, the rats in each group were sacrificed, and tissue was prepared for histological and immunohistochemistry analyses (*n* = 6), Western blot analyses (*n* = 6), and quantitative real-time PCR (qRT-PCR) measurements (*n* = 6).

### 4.4. Spinal Cord Compression Injury

The spinal cord was injured at vertebral thoracic level 10 (T_10_) using a previously established static compression model [41]. The rats were anesthetized using isoflurane (3% induction, 1.5% maintenance) in a mixture of 70% N_2_O and 30% O_2_ gas, and the skin was incised along the midline of the back, and the paravertebral muscles of the T_9_–T_11_ were dissected out. Body temperature was kept constant at 37 °C during the entire surgical procedure. The spinal cord at the T_10_ level was exposed by laminectomy, keeping the dura intact. The T_9_ and T_11_ spinal processes were clamped to prevent movements of the spinal cord, and compression was applied by suspending the base of the compression platform (area, 2 mm × 3 mm) onto the exposed spinal cord under microscopic control. A weight of 35 g was then applied statically to the platform for 5 min. The muscle layers were then sutured, and the skin layers were closed with wound clips. After surgery, the animals were allowed to recover from the anesthesia on a warm pad. Sham animals were only subjected to laminectomy without compression. Animals that exhibited abnormal post-operative condition were excluded from the data collection.

### 4.5. Behavioral Assessment

The behavioral recovery of the hindlimbs from motor disturbances after SCI was assessed using the Basso, Beattie, and Bresnahan (BBB) test [42]; the inclined plane test [43]; and the toe spread and hind foot bar grab tests [44]. All behavioral motor functions were assessed by another three examiners in a blinded manner separately with histology and molecular works. The scores of each test were evaluated at 1, 4, 7, 10, and 14 days after SCI for each animal in the same sequence with a resting time of 10 min between each test. At each test session, three separate measurements were made for each animal, and the mean of the three was considered a single score for that animal. 

#### 4.5.1. BBB Test

The rat was placed in an open field box (150 cm long, 120 cm wide, and 30 cm high) with a smooth floor, and two independent examiners studied its locomotor ability. Rats were tested in pairs for 4 min. During open field testing, rats were encouraged to locomote continuously. All hindlimb movements were recorded except for those that were a part of a reflex or were elicited by a contact with an examiner or another animal. The BBB test describes hindlimb joint movement, hindpaw placement, and forelimb-hindlimb coordination according to a scale from 0 (complete paralysis) to 21 (complete mobility) [42].

#### 4.5.2. Inclined Plane Test

The test consisted of measuring the maximum angle at which an animal could support its body weight for at least five seconds on an inclined board. The rats were placed facing downwards on an inclined board covered with a rubber mat. The board was raised in 5° increments during the process, and the maximum angle (0° to 90°) at which the rat could maintain its position was recorded [43].

#### 4.5.3. Toe Spread and Hind Foot Bar Grab Tests

In addition, tests for functional deficits after SCI were performed by scoring the toe spread and hind foot bar grab as described previously [44]. Toe spread is a reflex to spread the toes elicited by lifting the rat. The rat was placed on a table, it was held by the body with one hand, and lifted with its legs allowed to hang free. The amount of toe spreading in each hindlimb was graded individually as follows: 0, no spreading; 1, minimum spreading with flaccid fingers; 2, partial spreading; 3, normal full toe spread. Hind foot bar grab is also a reflex in response to contact of the hind foot to a small diameter bar. The scores of hind foot bar grab test were graded as follows: 0, no response when the hind foot touches the bar; 1, the hind foot responds to a contact with the bar, but cannot grab the bar; 2, the hind foot grabs the bar, but weakly; 3, the hind foot successfully grabs the bar and pulls it against its body.

### 4.6. Spinal Cord Tissue Preparation and Histological Assessment

Rats were deeply anesthetized and perfused transcardially with 0.05 M phosphate-buffered saline (PBS) containing 4% paraformaldehyde. The spinal cord was removed and post-fixed in the same perfusing solution overnight at 4 °C. Then, the spinal cord was cut into two pieces corresponding to rostral and caudal parts (1 cm length each) including the injury epicenter. The two pieces of spinal tissue were mounted in a same block and transverse 20 μm thick sections were made serially using a freezing microtome (Leica, 2800 N, Nussloch, Germany) from the injury epicenter. For histological assessment, the spinal cord sections were stained with cresyl violet (CV) solution and Luxol fast blue (LFB) solution using sections at 500 μm intervals. The lesion area and spared neurons were analyzed using ImageJ software (Ver. 1.44p, NIH, Bethesda, MD, USA). Lesion areas were analyzed on LFB-stained sections at 2 mm intervals. Spared neurons in the ventral horn were counted on CV-stained sections in the epicenter and on the rostral and caudal sections present 4 mm apart from the lesion epicenter. The number of spared neurons was normalized using the same area (50,000 μm^2^).

### 4.7. Immunoshistochemistry

Spinal sections were stained with free-floating 3,3′-diaminobenzidine tetrahydrochloride (DAB). The sections were rinsed with 0.05 M PBS and incubated in 1% hydrogen peroxide in PBS at room temperature for 15 min. The sections were then incubated with primary antibodies against Bax (1:200, ab7977, Abcam), iNOS (1:200, #610329, BD), COX-2 (1:200, #160106, Cayman), and Iba1 (1:500, #019-19741, Wako) at 4 °C overnight. After washing with PBS three times, they were then incubated with biotinylated anti-rat secondary antibody (1:200, Millipore, Billerica, MA, USA) at room temperature for 1 h. After rinsing three times with PBS, they were subjected to avidin-biotin complex (Vector Laboratories, Burlingame, CA, USA) with peroxidase coupling in a mixture containing 0.05% DAB (Sigma-Aldrich, St. Louis, MO, USA) and 0.03% H_2_O_2_ for 2–5 min. Counter-staining was performed lightly using 0.2% methyl green solution or 0.1% CV solution. Images of DAB-stained brain sections were captured using a light microscope (BX51, Olympus, Tokyo, Japan) equipped with CCD camera (DP70, Olympus).

### 4.8. Quantitative Real-Time PCR Measurement

Pro-inflammatory cytokines (TNF-α, IL-1β, IL-6) in the spinal cord tissue were measured using the qRT-PCR method. The rats were sacrificed by decapitation and the spinal cord tissue was rapidly dissected. Total RNA was extracted from the samples using Trizol (Qiagen, Hilden, Germany), according to the manufacturer’s protocol. One microgram of total RNA was transcribed into DNA by using iScript cDNA synthesis Kit (Bio-Rad, Hercules, CA, USA). After reverse transcription, qRT-PCR was performed using a pre-optimized primer/probe mixture with iQ SYBR Green Supermix kit (Bio-Rad) and the CFX 96 REAL-TIME PCR Detection System (Bio-Rad). Primer sequences for the genes analyzed are as follows: (1) TNF-α; forward, 5′-TGAGAAGTTCCCAAATGGC-3′; reverse 5′-GCTACAGGCTTGTCACTC-3′; (2) IL-1β; forward, 5′-TGAGCACCTTCTTTTCCTTCA-3′; reverse; 5′-TTGTCTAATGGGAACGTCACAC-3′; (3) IL-6; forward, 5′-AGACTTCACAGAGGATACCA-3′; reverse, 5′-GCATCATCGTTGTTCATACA-3′; (4) β-actin; forward, 5′-TTTCCAGCCTTCCTTGGGTATG-3′; reverse, 5′-CACTGTGTTGGCATAGAGGTCTTTAC-3′. The relative difference in the expression between the samples was represented using cycle time values normalized using measurements of the housekeeping gene β-actin as a reference. The sample values represent x-fold differences from a normal sample (given a value of 1) within the same experiment.

### 4.9. Western Blotting

Bax, Bcl-2, iNOS, COX-2, and Iba1 expression levels in the spinal tissue were measured using the western blotting method. The spinal tissue of approximately 2 cm in length including the injury epicenter, was homogenized and sonicated on ice in lysis buffer (50 mM Tris-HCl, pH 8.0, 150 mM NaCl, 1% Triton X-100, 0.5% sodium deoxycholate, 0.1% sodium dodecyl sulfate (SDS), 1 mM EDTA, 1% protease inhibitor cocktail; Sigma). After centrifugation, the supernatant was collected and the protein concentration was determined using the Bradford method. Lysate samples containing 50 mg of protein were fractionated by 10% SDS-polyacrylamide gel electrophoresis and then subjected to Western blot analysis. The primary antibodies used in this study were rabbit anti-Iba1 antibody (#016-20001, Wako, Japan) and mouse anti-β-actin antibody (Chemicon, Temecula, CA, USA).

### 4.10. Statistical Analysis

All data in this study are presented as mean ± standard error of the mean. They were analyzed using Student’s *t*-test. The statistical significance was set at *p* < 0.05.

## 5. Conclusions

In conclusion, this study demonstrates that GRg3 effectively promoted the recovery of motor function, ameliorated spinal tissue damage, attenuated neuronal apoptosis and the overproduction of pro-inflammatory mediators, and suppressed microglial activation after SCI. Therefore, this study suggests that GRg3 can be a candidate for the development of a therapeutic agent for SCI.

## Supplementary Materials

Supplementary materials can be accessed at: http://www.mdpi.com/1420-3049/22/1/122/s1.
