# Supplementary material for: Ginsenoside Rg3 Improves Recovery from Spinal Cord Injury in Rats via Suppression of Neuronal Apoptosis, Pro-Inflammatory Mediators, and Microglial Activation
Source: Molecules. 2017 Jan 12;22(1):122. doi: 10.3390/molecules22010122 (PMC6155773; doi:10.3390/molecules22010122)
Supplement: Supplementary file 1 [file molecules-22-00122-s001.pdf]

Supplementary Materials: Ginsenoside Rg3 Improves Recovery from Spinal Cord Injury in Rats via Suppression of Neuronal Apoptosis, Pro-Inflammatory Mediators, and Microglial Activation

Dong-Kyu Kim, Ki-Jung Kweon, Pyungsoo Kim, Hee-Jung Kim, Sung-Soo Kim, Nak-Won Sohn, Sungho Maeng and Jung-Won Shin

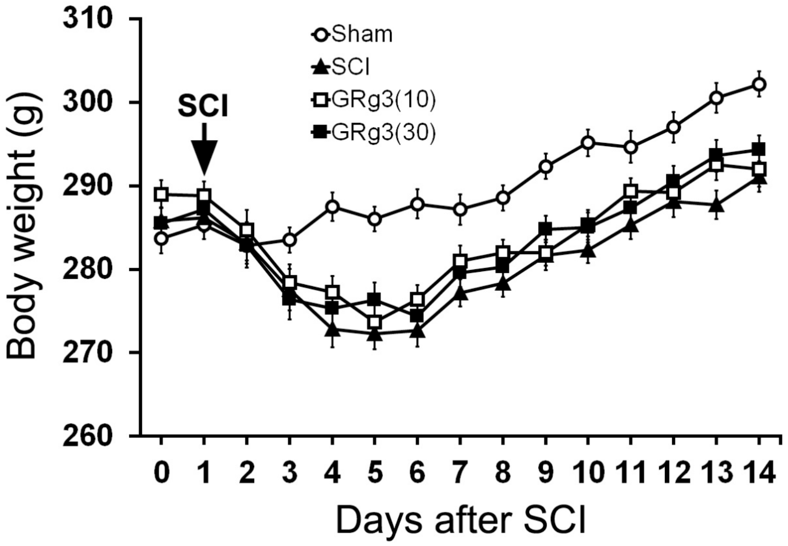

Figure S1. Changes in body weight of rats subjected to spinal cord injury (SCI). The body weight of all rats in the SCI, GRg3(10), and GRg3(30) groups decreased significantly during first 3 days after SCI. Data are represented as mean ± SEM (n = 18 in each group).
